# Rescue of long-term memory after reconsolidation blockade

**DOI:** 10.1038/ncomms8897

**Published:** 2015-08-04

**Authors:** Simon Trent, Philip Barnes, Jeremy Hall, Kerrie L. Thomas

**Affiliations:** 1 Neuroscience and Mental Health Research Institute, Cardiff University, Haydn Ellis Building, Maindy Road, Cardiff CF24 4HQ, UK; 2 Research and Innovations Services, Cardiff University, 30-36 Newport Road, Cardiff CF24 0ED, UK; 3 MRC Centre for Neuropsychiatric Genetics and Genomics, Haydn Ellis Building, Cardiff University, Maindy Road, Cardiff CF24 4HQ, UK; 4 School of Biosciences, Cardiff University, Museum Avenue, Cardiff CF10 3AX, UK

## Abstract

Memory reconsolidation is considered to be the process whereby stored memories become labile on recall, allowing updating. Blocking the restabilization of a memory during reconsolidation is held to result in a permanent amnesia. The targeted knockdown of either *Zif268* or *Arc* levels in the brain, and inhibition of protein synthesis, after a brief recall results in a non-recoverable retrograde amnesia, known as reconsolidation blockade. These experimental manipulations are seen as key proof for the existence of reconsolidation. However, here we demonstrate that despite disrupting the molecular correlates of reconsolidation in the hippocampus, rodents are still able to recover contextual memories. Our results challenge the view that reconsolidation is a separate memory process and instead suggest that the molecular events activated initially at recall act to constrain premature extinction.

## Abstract (web summary)

Recalled memories enter a labile state and are thought to be restabilized through reconsolidation. Here, the authors challenge the long-held notion that reconsolidation is a distinct memory process and demonstrate that the molecular events initiated at recall act instead to constrain premature extinction.

## Graphical abstract

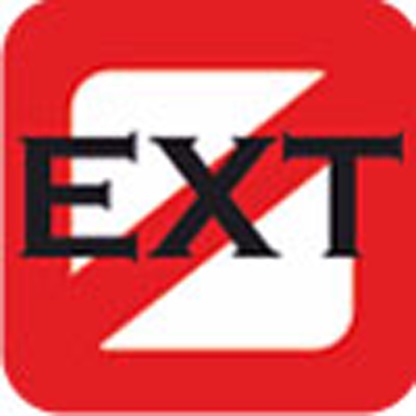

Reconsolidation theory proposes that stable, consolidated memories under certain experimental conditions can enter into a labile state on recall, allowing the updating or strengthening of the stored memory^1^,^2^. These memories must then be actively restabilized through a process of reconsolidation otherwise they are lost, resulting in a permanent amnesia^3^,^4^. Memory recall is accompanied by specific molecular processes, including the expression of the genes *Zif268* and *Arc* and the synthesis of new proteins. Disruption of these molecular processes following memory recall in conditioning studies has been shown to result in a permanent amnesia, and this is considered to be the key experimental demonstration of the existence of reconsolidation as a separate memory process^4^,^5^.

On the other hand, memory recall also has the potential to initiate extinction of the memory^6^. Extinction is considered to be a new learning process in which stimuli gain new predictive properties. As such, extinction memories come to compete for behavioural control at recall and the behaviour driven by the original memory is suppressed^7^,^8^. Notably, following extinction, memory-driven behaviours can later be revealed by experimental manipulations such as a reminder stimulus^9^. In conditioning studies, extinction is engaged when a conditioned association is repeatedly retrieved or recalled for an extended period without further reinforcement^10^,^11^. During recall, a careful balance must be achieved in maintaining the original memory and initiating extinction. It is therefore possible that the molecular processes accompanying the initial phase of memory recall act to constrain the premature engagement of extinction rather than to mediate reconsolidation.

In our contextual fear conditioning protocol in rats, targeted knockdown of plasticity and memory-associated molecules in the dorsal hippocampus during a brief recall trial resulted in the loss of expression of fear memory^5^. We sought to disambiguate between two alternative explanations of the role of the molecular events accompanying early memory recall (mediating reconsolidation versus constraining extinction) by the use of a reminder stimulus. The reminder stimulus was a low intensity unconditioned stimulus (US), below the intensity required to produce *de novo* conditioning (Supplementary Fig. 1)^12^,^13^. The expression of a fear memory after extinction should be reinstated by such a reminder US^9^; however, an experimental manipulation that disrupts reconsolidation should result in a permanent amnesia^4^, and as a consequence, the memory should not be recoverable by a reminder.

Here, we show that memory recall targeted knockdown of protein synthesis and the expression of reconsolidation-associated molecules Zif268 and Arc with directly infused antisense oligodeoxynucleotide (ASO) agents that are used routinely to define reconsolidation blockade, do not result in a permanent amnesia because fear memory can be recovered after a reminder US. These results call us to reassess the phenomena of memory reconsolidation as a constructive, independent mnemonic process and instead suggest that an active process at recall in the dorsal hippocampus acts to constrain the extinction of memories, rather than to facilitate reconsolidation.

## Results

**Figure 1:**
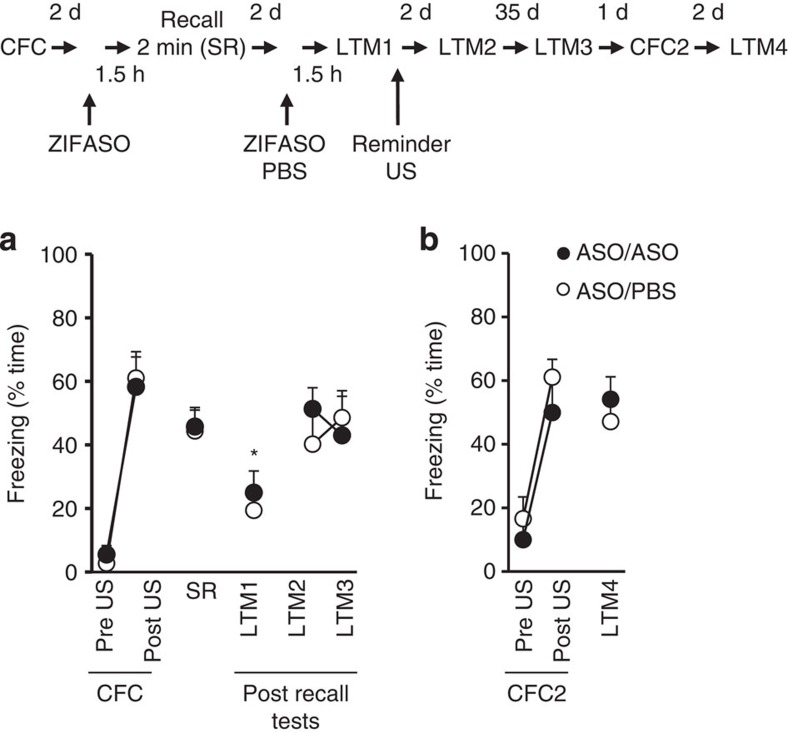
Intrahippocampal ZIFASO infusions before recall produce an impairment in CFM that can be reinstated by a reminder US. All rats were contextually fear conditioned (CFC) using a 2 s, 0.5 mA footshock (US) 2 min after placement into a novel context for 3 min. Two days later they received ZIFASO 90 min before a short 2 min recall session (SR). Rats received either PBS (ASO/PBS) or ZIFASO (ASO/ASO) 90 min before a 2 min long-term memory test (LTM) which co-terminated with a 2 s, 0.25 mA reminder US (LTM1). Tests were given 2 (LTM2) and 35 (LTM3) days later. All rats were subsequently conditioned (CFC2, 2 s, 0.5 mA) and tested (LTM4) in a novel context. (**a**) ZIFASO given prior to SR caused a reduction in conditioned freezing at LTM1 that returned to preinfusion levels after the reminder US and was maintained for at least 37 days (test; F_(3.277,32.770)_=18.910, *P*=0.000, *ɛ*=0.655, repeated measures ANOVA). This recovery was seen despite further ZIFASO infusions before LTM1 to block memory strengthening (test × infusion; F_(3.277,32.770)_=0.411, *P*=0.839, *ɛ*=0.655, repeated measures ANOVA). (**b**) No fear memory was seen in a novel context prior to a second 2 s, 0.5 mA US (CFC2) and post US plus conditioned freezing at recall (LTM4) indicates intact hippocampal function in the infused rats (test × infusion; F_(1.797,17.969)_=2.054, *P*=0.154, *ɛ*=0.898, repeated measures ANOVA). *P<0.05 (corrected for multiple comparisons, ANOVA) compared to SR and LTM2. *n*=6 per group. Results are shown as mean±s.e.m. d, days.

The transcription factor *Zif268* is rapidly expressed following memory recall^14^ and has been argued to be a key molecular correlate of reconsolidation^5^. We have previously shown that hippocampal infusion of ASO to *Zif268* during a 2-min short recall (SR) session results in the loss of a recently acquired contextual fear memory (CFM). Notably, the *Zif268*-specific loss of CFM was robust and persistent and did not undergo spontaneous or state-dependent recovery suggesting a permanent amnesia consistent with a role of *Zif268* in reconsolidation^5^. However, we now show that CFM can be recovered after ZIFASO infusion by interposing a reminder stimulus at a subsequent recall trial (Fig. 1). The recovery of the conditioned response (CR) by the reminder was not due to the strengthening of the residual memory, a *Zif268*-dependent process^15^, because reinstatement was also seen when ZIFASO was administered before the reminder session. Also, reinstatement of the CR by the reminder cue occurred when ZIFASO alone or when combined infusions of ZIFASO and BDNFASO were administered before the reminder US (Supplementary Fig. 2a,b), demonstrating that the recovery of the CR was not due to additional incremental learning by the reminder US^16^, because BDNFASO blocks new learning in the absence^5^ and presence of ZIFASO (Supplementary Fig. 2c). In addition, the ASO infusions are unlikely to be causing non-specific effects in the hippocampus because retrieval of CFM is intact. Later, the rats could discriminate between the conditioned context and a new context, and could support this conditioning to a new context (Fig. 1a,b) and there is also no evidence of gross damage to the dorsal hippocampus after histological examination^17^. Thus, the experimental manipulation is consistent with the ZIFASO targeting extinction, therefore, implicating Zif268 to act on constraining premature extinction, opposed to mediating reconsolidation.

**Figure 2:**
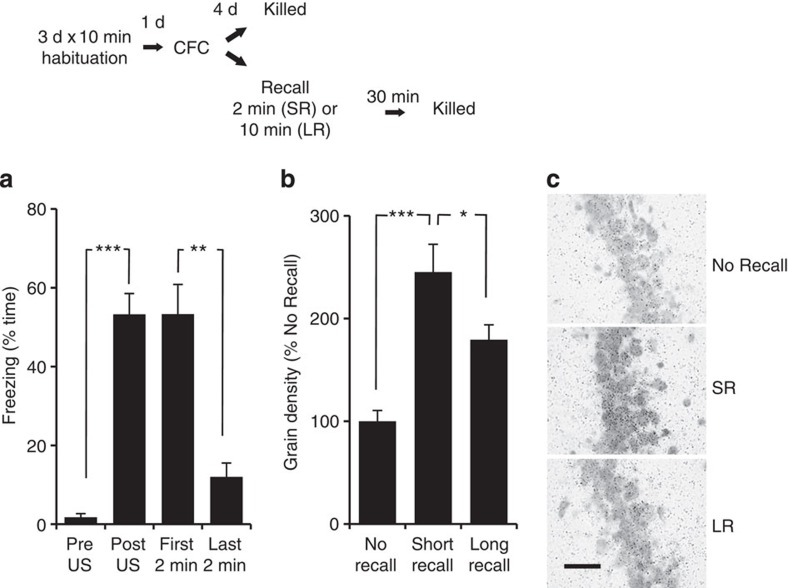
*Arc* expression in the CA1 increases with recall (SR and longer re-exposure (LR)), with the greatest increase at SR. Rats (*n*=30) were habituated to a novel context for 10 min/d for 3 d prior to conditioning. Recall of the fear memory 4 d later consisted of exposing rats to the conditioned context (CS) for either 2 min (SR, short recall, *n*=10) or 10 min (LR, long recall, *n*=10). Control rats (*n*=10) did not undergo recall (no recall). (**a**) There was as an increase in freezing behaviour post US during the conditioning period, but no TEST × GROUP interaction (F_(2,27)_=0.274, *P*=0.763, repeated measures ANOVA). The LR group showed a reduction in freezing during the last 2 min of the recall session indicating a robust within session extinction. (**b**) *In situ* hybridization revealed the regulation of *Arc* expression in CA1 30 min after recall (F_(2,27)_=15.145, *P*=0.000, ANOVA). This was due to an increase in the expression in the SR and LR groups, with a larger increase correlated with SR. *P<0.05, **P<0.01 and ***P<0.001 compared with the no recall control (Tukey's test). (**c**) Photomicrographs (100 × ) of small dark silver grains (an index of *Arc* expression) associated with CA1 pyramidal cells. Thus, the duration of the exposure to a conditioned context results in the differential regulation of *Arc* in hippocampal CA1. Scale bar, 50 μM. Results are shown as the mean±s.e.m. d, days.

**Figure 3:**
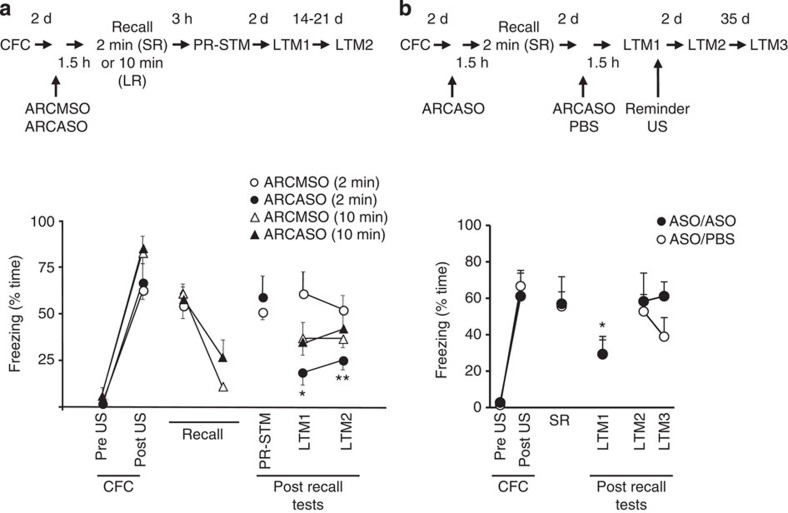
Intrahippocampal ARCASO infusions before recall produce impairment in CFM that can be reinstated by a reminder US. (**a**) Rats were exposed to the conditioning context for 2 min (*n*=10) or 10 min (*n*=12) 2 d after CFC. Half the rats received ARCMSO or ARCASO 1.5 h before recall. There was no difference in conditioned fear between the groups during the first 2 min of the extinction training (F_(1,10)_=0.849, *P*=0.379, repeated measures ANOVA) and no effect of ARCASO on within-session extinction (F_(1,10)_=3.789, *P*=0.08, repeated measures ANOVA). ARCASO prior to recall had an effect on freezing measured during the recall tests (first 2-min extinction training, PR-STM, LTM1 and LTM2) in the 2-min recall group (test × infusion; F_(2.084,16.674)_=7.466, *P*<0.001, *ɛ*=0.695, repeated measures ANOVA), but not the 10-min group (TEST (first 2-min extinction training, LTM1 and LTM2) X INFUSION; F_(1.713,17.128)_=0.844, *P*=0.445, *ɛ*=0.856, repeated measures ANOVA). This manifested as a reduction in conditioned fear in the ARCASO compared with the ARCMSO administered rats at LTM1 and LTM2 (*P<0.05, **P<0.01, Tukey's test). (**b**) Rats received ARCASO 1.5 h before a short 2-min recall session (SR) 2 d after CFC. Subsequently, ARCASO (ASO/ASO, *n*=6) or ARCMSO (ASO/MSO, *n*=6) was administered 1.5 h before a 2-min recall session that co-terminated with a 2 s, 0.25 mA reminder US (LTM1). Compared with SR, a reduction in conditioned freezing was seen at LTM1 before the reminder demonstrating that ARCASO infusions reduced fear behaviour and there was no difference in the responses between the two groups (test × infusion; F_(3.179,31.788)_=1.019, *P*=0.417, *ɛ*=0.636, repeated measures ANOVA). The reminder US reinstated contextual fear to preinfusion levels 2 and 37 days later (LTM2 and LTM3). *P<0.05 (corrected for multiple comparisons, ANOVA) compared with SR and LTM2. Results are shown as the mean±s.e.m. d, days.

Arc (Activity-regulated cytoskeletal-associated protein) has also been argued to play an essential role in fear memory reconsolidation in the amygdala^18^. Here we confirm that *Arc* expression is rapidly increased in the hippocampal CA1 region after the recall of CFM, in agreement with other studies^19^,^20^,^21^ (Fig. 2a–c). Furthermore, the increase in *Arc* expression was greater following the SR session compared with a longer 10-min re-exposure to the conditioned context (Fig. 2a–c), suggesting the presence of a time-limited refractory period for *Arc* expression in response to increasing context exposure, consistent with the findings of others^22^,^23^,^24^. The duration of recall is significant as a 2-min recall session does not appreciably affect conditioned freezing, but renders the CFM susceptible to disruption^5^, while 10-min recall produces extinction^17^. Infusing ARCASO into the dorsal hippocampus before SR resulted in a long-lasting decrease in fear memory (Fig. 3a), consistent with the proposed role of Arc in reconsolidation. This effect of the ARCASO was selective because the extinction associated with longer re-exposure was apparently unaffected by ARCASO. The efficacy of the ARCASO was confirmed by western blot by showing a sustained reduction of Arc protein expression in the CA1 following intrahippocampal ARCASO infusions (Supplementary Fig. 3). Furthermore, the decrease was confined to the CA1 and not to dorsal dg/CA3 samples from the same rats (Supplementary Fig. 4). This shows that the effects of the ARCASO were confined to the target site of the infusions and the ability of the ARCASO to disrupt the behavioural expression of fear memory is correlated with reduced Arc in CA1 specifically.

To differentiate the effects of ARCASO in either blocking reconsolidation or facilitating extinction with SR, we again used a brief reminder US. This manipulation revealed that after ARCASO treatment, the reminder US reinstated memory during recall (Fig. 3b). The reinstatement of the memory is seen when ARCASO or PBS is given before the SR (Fig. 3a), demonstrating that neither state-dependent retrieval deficits^25^ or new learning^26^ underlie the recovery of the CR. Therefore, the profile of ARCASO effects on the CR, like that of ZIFASO, is consistent with Arc playing a role in preventing extinction rather than in mediating reconsolidation. We additionally ruled out the requirement for the combined activities of Zif268 and Arc in reconsolidation via the co-infusion of ZIFASO and ARCASO before SR (Supplementary Fig. 5).

**Figure 4:**
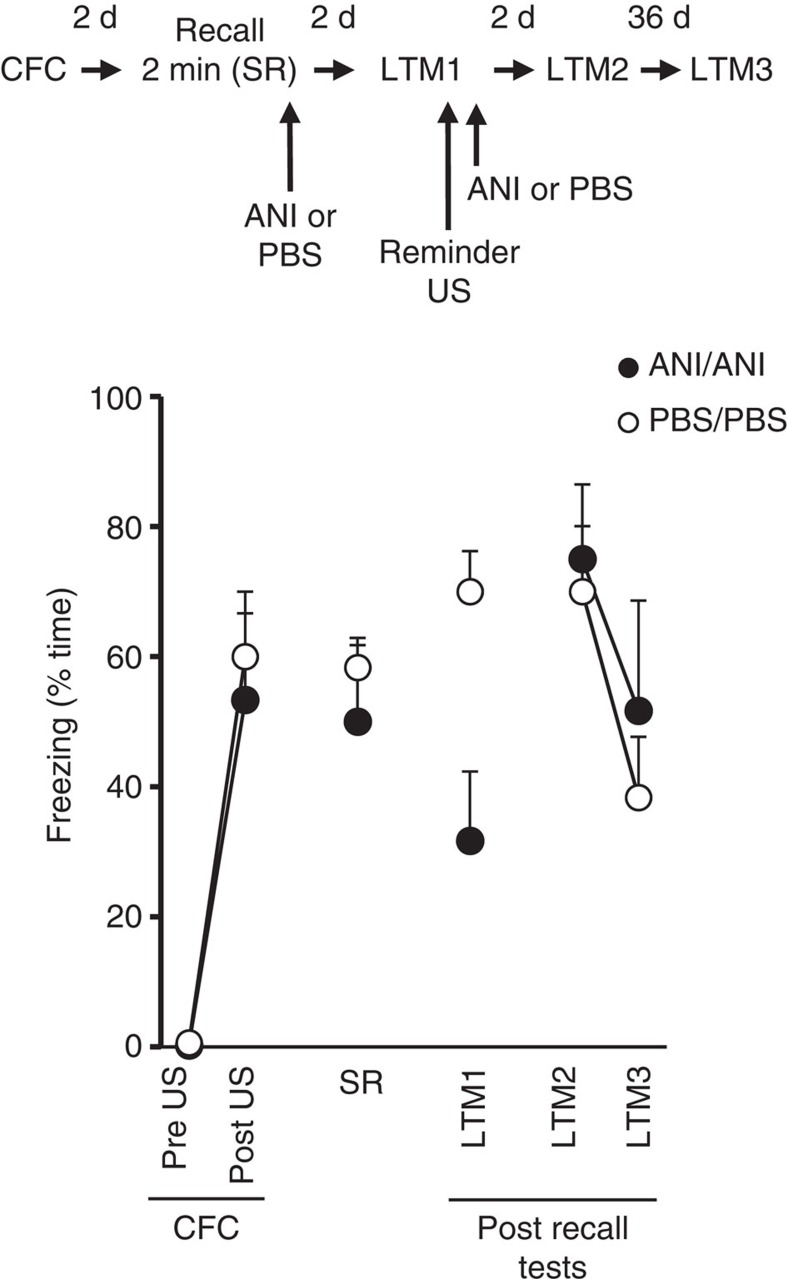
Anisomycin infused immediately after recall was insufficient to permanently impair CFM because reinstatement by a reminder US was observed. All rats received intrahippocampal infusion of either anisomycin (ANI) or PBS immediately after a short 2 min recall session (SR) 2 d after CFC, followed by a further round of infusions after a CFM test which co-terminated with a 2 s, 0.25 mA reminder US 2 d later (LTM1, *n*=5 for ANI/ANI and PBS/PBS groups). Anisomycin after SR impairs CFM at LTM1 (test × infusion; F_(1,8)_=9.521, *P*=0.015, repeated measures ANOVA). Recovery of CR was observed by the reminder US, despite additional anisomycin infusions to prevent residual learning or memory strengthening, which was demonstrated by the lack of group differences at LTM2 and LTM3 tests 2 and 38 d later (F_(1,8)_=0.409, *P*=0.540, repeated measures ANOVA). Results are shown as mean±s.e.m. d, days.

A key experimental underpinning of reconsolidation theory is the finding that blockade of protein synthesis following memory recall by anisomycin leads to a permanent amnesia^4^,^5^. This has been considered as proof that protein synthesis-dependent restabilization processes following recall-induced labilization are necessary for the memory to persist^27^. Using identical experimental manipulations to those we have used before^5^, we confirmed that anisomycin infused into the dorsal hippocampus after a SR trial produced the expected reduction in conditioned freezing at later test, consistent with previous studies (Fig. 4). However, we now show that the CFM can be subsequently recovered following the administration of a reminder US. The recovery of the CR after reminder is seen when anisomycin is additionally infused after the reminder US. This excludes the possibility that the initial reduction in CR derives from a retrieval failure via state-dependent mechanisms^25^, or is a result of new learning, strengthening or updating of the memory^5^,^15^. Anisomycin-treated animals also remain capable of fear learning in a new context, precluding any permanent damage to the hippocampus (Supplementary Fig. 6). Since a canonical characteristic of extinction is that the CR is temporarily suppressed but returns under various conditions, the reminder-induced return of CFM thus reveals an active, protein synthesis-dependent process in the hippocampus during SR that opposes extinction rather than mediating reconsolidation.

## Discussion

In conclusion, the observations that CFM can be recovered after using agents that are used routinely to define reconsolidation blockade calls us to reassess the phenomena of memory reconsolidation as a constructive, independent mnemonic process. Indeed, the temporary nature of CFM deficits by protein synthesis inhibitors at recall has been previously reported^28^,^29^. We add further, weight to these studies by showing recovery despite disrupting specific molecular correlates of reconsolidation. In addition these results are corroborated by human studies that have shown little evidence for the reconsolidation of hippocampal-dependent memories after retrieval^30^,^31^,^32^. Although we cannot exclude the possibility that reconsolidation may exist for other kinds of associative memory such as amygdala-based emotional memory^4^, a greater systematic investigative approach will be required in future when rejecting extinction-based interpretations in favour of reconsolidation-based views of recall-induced amnesia by different experimental interventions. A single example of memory rescue in such cases favours an extinction-based interpretation of the phenomena.

Our results suggest that when memory is recalled, a dynamic balance exists between maintaining the relevance of the original memory and extinction. We show that under conditions of short-term recall, *Zif268*- and *Arc*-dependent cellular processes in the hippocampus act to constrain extinction rather than to mediate reconsolidation. Under this model, changes in the expression of genes such as *Zif268* and *Arc* in an early phase of recall blocks the premature engagement of extinction. Consequently, inhibiting the expression of these genes manifests as a more rapid extinction process that can then later be recovered by a reminder. However, under recall conditions that favour extinction, such as longer recall, these molecular inhibitors of extinction are inactive and extinction can proceed. As a consequence of identifying an active molecular process that prevents the extinction of memory, a simpler theoretical framework can be drawn on when assessing the site of action of treatments that influence memory expression; either by directly impacting on extinction or on the regulatory mechanisms that control extinction.

Reconsolidation is seen as an adaptive mechanism for maintaining memory relevance via updating or strengthening^2^. Furthermore, it is often held as a universal property of associative memories and failure to observe reconsolidation experimentally, termed ‘reconsolidation resistance', have been explained away because the ‘boundary' conditions to engage it were not exceeded, or were altered by experience via metaplastic processes at the network level^33^. In fact, the two key boundary conditions proposed for reconsolidation, the *un*predictability of the retrieval stimulus^34^ and the presence of novel information^35^,^36^,^37^ are those that give rise to new learning including extinction^7^. Our experimental demonstration of reconsolidation blockade as facilitated extinction suggests that there is no competition between memory updating or strengthening (functions attributed to reconsolidation) and extinction, which are all described by similar learning rules, but rather it may suggest a molecular and cellular process that prevents new learning, which is gated by the similarity of the recalled memory to on-going experience^38^. In this regard, we can now focus on investigating the boundary conditions for extinction. Our data and the observation of the susceptibility of memory to disruption without retrieval (for example, refs 39, 40) are also more consistent with lingering consolidation, dynamic trace and comparator interpretations of memory^41^,^42^,^43^,^44^,^45^,^46^ rather than a recall-based reconsolidation view.

In summary, we demonstrate the recovery of contextual memories despite disrupting the molecular correlates of reconsolidation in the hippocampus. By doing so, we question the process of reconsolidation as a separate mnemonic memory process and suggest that the molecular events activated initially at recall act to constrain premature extinction. Instead, our results suggest that when memory is recalled, a dynamic balance exists between maintaining or strengthening the original memory and extinction.

## Methods

### Subjects

The subjects were adult male Lister hooded rats weighing 280–350 g. They were housed in pairs, in holding rooms maintained at 21 °C on a reversed-light cycle (12-h light/dark; lights on at 2200 h). All experiments were conducted in the dark period of the rats. Food and water were freely available throughout the experiment. All procedures were conducted in accordance with local Cardiff University's Animal Welfare and Ethical Review Body approval and the United Kingdom 1986 Animals (Scientific Procedures) Act (Project license PPLs 30/2236 and 30/2722). Sample size calculations were performed in http://www.stat.uiowa.edu/~rlenth/Power. All CFC rats were included in the final data analyses unless explicitly noted.

### Surgery and microinfusions into the dorsal hippocampus

Steel double guide cannula aimed at the dorsal hippocampus (AP-3.50, relative to bregma) were surgically implanted under anaesthesia^47^ at least 1 week before behavioural training. Bilateral infusions via the chronically indwelling cannula were carried out in awake rats using a syringe pump, connected to injectors (28 gauge, projecting 1 mm beyond the guide cannula) by polyethylene tubing. Anisomycin (Sigma) was prepared at 80 μg μl^−1^ in sterile PBS (pH 7.4), while vehicle controls received PBS alone. ODNs were PAGE-purified phosphorothioate end-capped 18–20-mer sequences (SigmaGenosys): *Arc* antisense ODN, ARCASO, 5′-GTCCAGCTCCATCTGCTCGC-3′; *Arc* missense ODN, ARCMSO, 5′-CCACGCCATCGTGCCTTCGT-3′; *Zif268* antisense ODN, ZIFASO, 5′-GGTAGTTGTCCATGGTGG-3′ and *BDNF* antisense ODN, BDNFASO, 5′-TCTTCCCCTTTTAATGGT-3′. All ODN sequences were subjected to a BLAST search on the National Center for Biotechnology Information BLAST server using the Genbank database. The antisense sequence had positive matches only for its target mRNA sequence, and no other rat or human-coding sequences. The control missense sequence, which included the same 20 nucleotides as the cogent ASO sequence but in a scrambled order, did not generate any full matches to identified gene sequences in the database. All ODNs were resuspended in sterile PBS (pH 7.4), ARCASO and ARCMSO to a concentration of 2 nmol μl^−1^, while combined ASO infusions gave a final concentration of each of 2 nmol μl^−1^. The volume of ODN infusions was 1 μl per hemisphere delivered at a rate of 0.125 μl min^−1^ and were infused 90 min before either recall or LTM1 sessions. The volume of anisomycin/vehicle infusions was 1 μl per hemisphere, delivered at a rate of 0.5 μl min^−1^ and given immediately after either recall or LTM1. All trained rats, unless indicated, were included in subsequent analyses. Where the procedure permitted, histological determination of the cannula placement using β-thionin staining of Nissl substance showed that all the brains examined showed the placement of cannula to be in the dorsal hippocampus with minimal tissue damage or ventricle enlargement.

### Contextual fear conditioning

Conditioning was performed in one of two distinct contexts. During the 3-min conditioning training trial, rats received a single 2 s, 0.5 mA scrambled footshock (US) 2 min after being placed into the conditioning context. All rats were returned to the home cages after conditioning. Recall of CFM occurred 2–4 days later and consisted of exposing rats to the conditioned context for either 2 min (Short Recall, SR) or 10 min (Long Recall, longer re-exposure). Retrieval tests 3 h (post-retrieval short-term memory, PR-STM), or 2–39 days (long-term memory, LTM1- 3) after recall consisted of exposing the rat to the conditioning context for 2 min. In some experiments a 2 s, 0.25 mA scrambled footshock was given to co-terminate with the PR-STM or LTM1 as a reminder stimulus (Reminder US). Freezing behaviour served as a measure of the conditioned fear response (CR) to the context during the conditioning, extinction training and recall tests. This was video recorded and quantified by an observer blind to the experimental group. One unit of freezing was defined as a continuous absence of movement other than that required for respiration in 1 s sampled every 10 s. Freezing behaviour was analysed in a repeated measures analysis of variance (repeated measures ANOVA) with test as a within-subjects factor, or by ANOVA. For repeated measures ANOVA, Mauchly's Test of Sphericity was applied. If the sphericity assumption was not met, the Greenhouse–Geisser correction was applied. Tukey's test was then used for *post hoc* analysis to determine the sources of significance (*P<0.05, **P<0.01 and ***P<0.001). *Post hoc* planned comparisons were made using repeated measures ANOVA and the *P*-value constrained by the number of comparisons made.

### *In situ* hybridization

Thirty minutes after recall rats were killed by carbon dioxide inhalation and the brain was rapidly removed and processed for *in situ* hybridization of an oligonucleotide probe (5′-AGCATCTCAGCTCGGCACTTACCAATCTGCAGGATCACATTGGGT-3′, SigmaGenosys Ltd, Cambridge, UK) complementary to nucleotides 288–332 of *Arc* (NM 019361), 3′-end-labelled with [α-^35^S] dATP^17^. Images were captured using LeicaQWIN imaging software (Leica Microsystems (UK) Ltd, Milton Keynes, UK) using an × 100 objective under oil immersion. Silver grain density was assessed in dorsal hippocampal CA1 pyramidal cells (approximately bregma −3.3 to 3.944 mm) using ImageJ imaging software ( http://rsbweb.nih.gov/ij/). Grains (total and non-specific) were counted over sufficient randomly selected neurons from each region for each animal such that the SE of the counts for any region was <10% of the population mean (typically 24 cells). In each case, cells were selected from at least three non-adjacent separate sections. A specific grain count was then calculated for each region by subtracting total and non-specific counts. The mean silver grain count in each region for each animal was then divided by the mean count in that region for the No Recall control group to give a standardized grain count (%) for each group. Standardized results were analysed by ANOVA, and *post hoc* comparisons were made using Tukey's test.

### Western blotting

At 2 or 6 h after retrieval testing, rats were sacrificed by carbon dioxide inhalation. The rats were decapitated and the brain was rapidly removed and placed on ice. The hippocampal dentate gyrus/CA3 and CA1 regions were microdissected and the dorsal half was isolated and frozen on dry ice before storage at −80 °C. Tissue lysates and western blotting were performed essentially as previously described^47^. Primary and secondary antibodies were diluted in TBST containing 0.5% Tween 20 and 2% ECL Advance blocking agent (GE Healthcare Inc, Chalfont St Giles, UK) and they were used at the following concentrations: Arc (H-300 Santa Cruz Biotechnology Inc, Insight Biotechnology Ltd, Wembley, UK), 1:20,000; GAPDH (ab9485, Abcam plc, Cambridge, UK), 1:10,000 and goat anti-rabbit IgG (whole-molecule)-peroxidase conjugate (Sigma-Aldrich Company Ltd. Poole, UK), 1:10,000. Incubation in antibody solutions were all for 1 h at room temperature with the exception of blots that were incubated with Arc antibody, which were incubated at 4 °C, overnight. Blots were visualized using ECL Advance detection (GE Healthcare Inc, Chalfont St Giles, UK) and opposed to autoradiographic film. Autoradiographs of each western blot were developed to be linear in the range used for densitometry for each protein target and for GAPDH. Autoradiographic images were scanned, digitized and the amounts of Arc and GAPDH signal were quantified for each sample using ImageJ ( http://rsbweb.nih.gov/ij). Averaging the amount of GAPDH across samples on each western blot and deriving a normalization factor for each sample corrected for loading variation. Levels of Arc protein were standardized with respect to the mean measured in the No Recall control group. ANOVA was applied and Tukey's test was then used for *post hoc* analysis to determine the sources of significance.

## Additional information

**How to cite this article:** Trent, S. *et al.* Rescue of long-term memory after reconsolidation blockade. *Nat. Commun.* 6:7897 doi: 10.1038/ncomms8897 (2015).

## Supplementary Material

Supplementary Information Supplementary Figures 1-6

## References

[b1] Nader K. & Hardt O. A single standard for memory: the case for reconsolidation. Nat. Rev. Neurosci. 10, 224–234 (2009). 19229241 10.1038/nrn2590

[b2] Lee J. L. Reconsolidation: maintaining memory relevance. Trends Neurosci. 32, 413–420 (2009). 19640595 10.1016/j.tins.2009.05.002 PMC3650827

[b3] Misanin J. R., Miller R. R. & Lewis D. J. Retrograde amnesia produced by electroconvulsive shock after reactivation of a consolidated memory trace. Science 160, 554–555 (1968). 5689415 10.1126/science.160.3827.554

[b4] Nader K., Schafe G. E. & Le Doux J. E. Fear memories require protein synthesis in the amygdala for reconsolidation after retrieval. Nature 406, 722–726 (2000). 10963596 10.1038/35021052

[b5] Lee J. L., Everitt B. J. & Thomas K. L. Independent cellular processes for hippocampal memory consolidation and reconsolidation. Science 304, 839–843 (2004). 15073322 10.1126/science.1095760

[b6] Pavlov I. P. Conditioned reflexes: An investigation of the physiological activity of the cerebral cortex Oxford Univ. Press (1927). 10.5214/ans.0972-7531.1017309 PMC4116985 25205891

[b7] Rescorla R. A. & Wagner A. R. A Theory of Pavlovian conditioning: Variations in the Effectiveness Of Reinforcement and Nonreinforcement Appleton-Century-Crofts (1972).

[b8] Bouton M. E. Context, time, and memory retrieval in the interference paradigms of Pavlovian learning. Psychol. Bull. 114, 80–99 (1993). 8346330 10.1037/0033-2909.114.1.80

[b9] Myers K. M. & Davis M. Mechanisms of fear extinction. Mol. Psychiatry 12, 120–150 (2007). 17160066 10.1038/sj.mp.4001939

[b10] Eisenberg M., Kobilo T., Berman D. E. & Dudai Y. Stability of retrieved memory: inverse correlation with trace dominance. Science 301, 1102–1104 (2003). 12934010 10.1126/science.1086881

[b11] Suzuki A. *et al.* Memory reconsolidation and extinction have distinct temporal and biochemical signatures. J. Neurosci. 24, 4787–4795 (2004). 15152039 10.1523/JNEUROSCI.5491-03.2004 PMC6729467

[b12] Cai W. H., Blundell J., Han J., Greene R. W. & Powell C. M. Postreactivation glucocorticoids impair recall of established fear memory. J. Neurosci. 26, 9560–9566 (2006). 16971540 10.1523/JNEUROSCI.2397-06.2006 PMC3917138

[b13] Blundell J., Kouser M. & Powell C. M. Systemic inhibition of mammalian target of rapamycin inhibits fear memory reconsolidation. Neurobiol. Learn. Mem. 90, 28–35 (2008). 18316213 10.1016/j.nlm.2007.12.004 PMC2497420

[b14] Hall J., Thomas K. L. & Everitt B. J. Cellular imaging of zif268 expression in the hippocampus and amygdala during contextual and cued fear memory retrieval: selective activation of hippocampal CA1 neurons during the recall of contextual memories. J. Neurosci. 21, 2186–2193 (2001). 11245703 10.1523/JNEUROSCI.21-06-02186.2001 PMC6762622

[b15] Lee J. L. Memory reconsolidation mediates the strengthening of memories by additional learning. Nat. Neurosci. 11, 1264–1266 (2008). 18849987 10.1038/nn.2205

[b16] Gold P. E. & King R. A. Retrograde amnesia: storage failure versus retrieval failure. Psychol. Rev. 81, 465–469 (1974). 4474675 10.1037/h0036949

[b17] Kirtley A. & Thomas K. L. The exclusive induction of extinction is gated by BDNF. Learn. Mem. 17, 612–619 (2010). 21127000 10.1101/lm.1877010

[b18] Maddox S. A. & Schafe G. E. The activity-regulated cytoskeletal-associated protein (Arc/Arg3.1) is required for reconsolidation of a Pavlovian fear memory. J. Neurosci. 31, 7073–7082 (2011). 21562269 10.1523/JNEUROSCI.1120-11.2011 PMC3109861

[b19] Guzowski J. F., Setlow B., Wagner E. K. & McGaugh J. L. Experience-dependent gene expression in the rat hippocampus after spatial learning: a comparison of the immediate-early genes Arc, c-fos, and zif268. J. Neurosci. 21, 5089–5098 (2001). 11438584 10.1523/JNEUROSCI.21-14-05089.2001 PMC6762831

[b20] Zhang W. P., Guzowski J. F. & Thomas S. A. Mapping neuronal activation and the influence of adrenergic signaling during contextual memory retrieval. Learn. Mem. 12, 239–247 (2005). 15930502 10.1101/lm.90005 PMC1142451

[b21] Gusev P. A., Cui C., Alkon D. L. & Gubin A. N. Topography of Arc/Arg3.1 mRNA expression in the dorsal and ventral hippocampus induced by recent and remote spatial memory recall: dissociation of CA3 and CA1 activation. J. Neurosci. 25, 9384–9397 (2005). 16221847 10.1523/JNEUROSCI.0832-05.2005 PMC6725713

[b22] Guzowski J. F. *et al.* Recent behavioral history modifies coupling between cell activity and Arc gene transcription in hippocampal CA1 neurons. Proc. Natl Acad. Sci. USA 103, 1077–1082 (2006). 16415163 10.1073/pnas.0505519103 PMC1347968

[b23] Mamiya N. *et al.* Brain region-specific gene expression activation required for reconsolidation and extinction of contextual fear memory. J. Neurosci. 29, 402–413 (2009). 19144840 10.1523/JNEUROSCI.4639-08.2009 PMC6664934

[b24] Maddox S. A., Monsey M. S. & Schafe G. E. Early growth response gene 1 (Egr-1) is required for new and reactivated fear memories in the lateral amygdala. Learn. Mem. 18, 24–38 (2011). 21177377 10.1101/lm.1980211 PMC3023969

[b25] Overton D. A. State-dependent or "dissociated" learning produced with pentobarbital. J. Comp. Physiol. Psychol. 57, 3–12 (1964). 14125086 10.1037/h0048023

[b26] Guzowski J. F. *et al.* Inhibition of activity-dependent arc protein expression in the rat hippocampus impairs the maintenance of long-term potentiation and the consolidation of long-term memory. J. Neurosci. 20, 3993–4001 (2000). 10818134 10.1523/JNEUROSCI.20-11-03993.2000 PMC6772617

[b27] Ben Mamou C., Gamache K. & Nader K. NMDA receptors are critical for unleashing consolidated auditory fear memories. Nat. Neurosci. 9, 1237–1239 (2006). 16998481 10.1038/nn1778

[b28] Lattal K. M. & Abel T. Behavioral impairments caused by injections of the protein synthesis inhibitor anisomycin after contextual retrieval reverse with time. Proc. Natl Acad. Sci. USA 101, 4667–4672 (2004). 15070775 10.1073/pnas.0306546101 PMC384804

[b29] Power A. E., Berlau D. J., McGaugh J. L. & Steward O. Anisomycin infused into the hippocampus fails to block "reconsolidation" but impairs extinction: the role of re-exposure duration. Learn. Mem. 13, 27–34 (2006). 16452651 10.1101/lm.91206 PMC1360130

[b30] Kindt M., Soeter M. & Vervliet B. Beyond extinction: erasing human fear responses and preventing the return of fear. Nat. Neurosci. 12, 256–258 (2009). 19219038 10.1038/nn.2271

[b31] Soeter M. & Kindt M. Dissociating response systems: erasing fear from memory. Neurobiol. Learn. Mem. 94, 30–41 (2010). 20381628 10.1016/j.nlm.2010.03.004

[b32] Soeter M. & Kindt M. Disrupting reconsolidation: pharmacological and behavioral manipulations. Learn. Mem. 18, 357–366 (2011). 21576515 10.1101/lm.2148511

[b33] Finnie P. S. & Nader K. The role of metaplasticity mechanisms in regulating memory destabilization and reconsolidation. Neurosci. Biobehav. Rev. 36, 1667–1707 (2012). 22484475 10.1016/j.neubiorev.2012.03.008

[b34] Dudai Y. Reconsolidation: the advantage of being refocused. Curr. Opin. Neurobiol. 16, 174–178 (2006). 16563730 10.1016/j.conb.2006.03.010

[b35] Pedreira M. E., Perez-Cuesta L. M. & Maldonado H. Mismatch between what is expected and what actually occurs triggers memory reconsolidation or extinction. Learn. Mem. 11, 579–585 (2004). 15466312 10.1101/lm.76904 PMC523076

[b36] Rodriguez-Ortiz C. J., De la Cruz V., Gutierrez R. & Bermudez-Rattoni F. Protein synthesis underlies post-retrieval memory consolidation to a restricted degree only when updated information is obtained. Learn. Mem. 12, 533–537 (2005). 16166395 10.1101/lm.94505 PMC1240066

[b37] Morris R. G. *et al.* Memory reconsolidation: sensitivity of spatial memory to inhibition of protein synthesis in dorsal hippocampus during encoding and retrieval. Neuron 50, 479–489 (2006). 16675401 10.1016/j.neuron.2006.04.012

[b38] Osan R., Tort A. B. & Amaral O. B. A mismatch-based model for memory reconsolidation and extinction in attractor networks. PLoS ONE 6, e23113 (2011). 21826231 10.1371/journal.pone.0023113 PMC3149635

[b39] Rodriguez-Ortiz C. J., Balderas I., Garcia-DeLaTorre P. & Bermudez-Rattoni F. Taste aversion memory reconsolidation is independent of its retrieval. Neurobiol. Learn. Mem. 98, 215–219 (2012). 22910716 10.1016/j.nlm.2012.08.002

[b40] Shema R., Sacktor T. C. & Dudai Y. Rapid erasure of long-term memory associations in the cortex by an inhibitor of PKM zeta. Science 317, 951–953 (2007). 17702943 10.1126/science.1144334

[b41] Dudai Y. & Eisenberg M. Rites of passage of the engram: reconsolidation and the lingering consolidation hypothesis. Neuron 44, 93–100 (2004). 15450162 10.1016/j.neuron.2004.09.003

[b42] Alberini C. M. Mechanisms of memory stabilization: are consolidation and reconsolidation similar or distinct processes? Trends Neurosci. 28, 51–56 (2005). 15626497 10.1016/j.tins.2004.11.001

[b43] McKenzie S. & Eichenbaum H. Consolidation and reconsolidation: two lives of memories? Neuron 71, 224–233 (2011). 21791282 10.1016/j.neuron.2011.06.037 PMC3145971

[b44] Dudai Y. & Morris R. G. Memorable trends. Neuron 80, 742–750 (2013). 24183024 10.1016/j.neuron.2013.09.039

[b45] Hardt O., Nader K. & Nadel L. Decay happens: the role of active forgetting in memory. Trends Cogn. Sci. 17, 111–120 (2013). 23369831 10.1016/j.tics.2013.01.001

[b46] Stout S. C. & Miller R. R. Sometimes-competing retrieval (SOCR): a formalization of the comparator hypothesis. Psychol. Rev. 114, 759–783 (2007). 17638505 10.1037/0033-295X.114.3.759

[b47] Barnes P. & Thomas K. L. Proteolysis of proBDNF is a key regulator in the formation of memory. PLoS ONE 3, e3248 (2008). 18813339 10.1371/journal.pone.0003248 PMC2532744

