# Supplementary material for: Rescue of long-term memory after reconsolidation blockade
Source: Nat Commun. 2015 Aug 4;6:7897. doi: 10.1038/ncomms8897 (PMC4532853; doi:10.1038/ncomms8897)
Supplement: Supplementary Information — Supplementary Figures 1-6 [file ncomms8897-s1.pdf]

# Rescue of long-term memory after reconsolidation blockade

**Authors:** Simon Trent[1], Philip Barnes[2], Jeremy Hall[1, 3], Kerrie L. Thomas[1, 4]*

## Supplementary Information

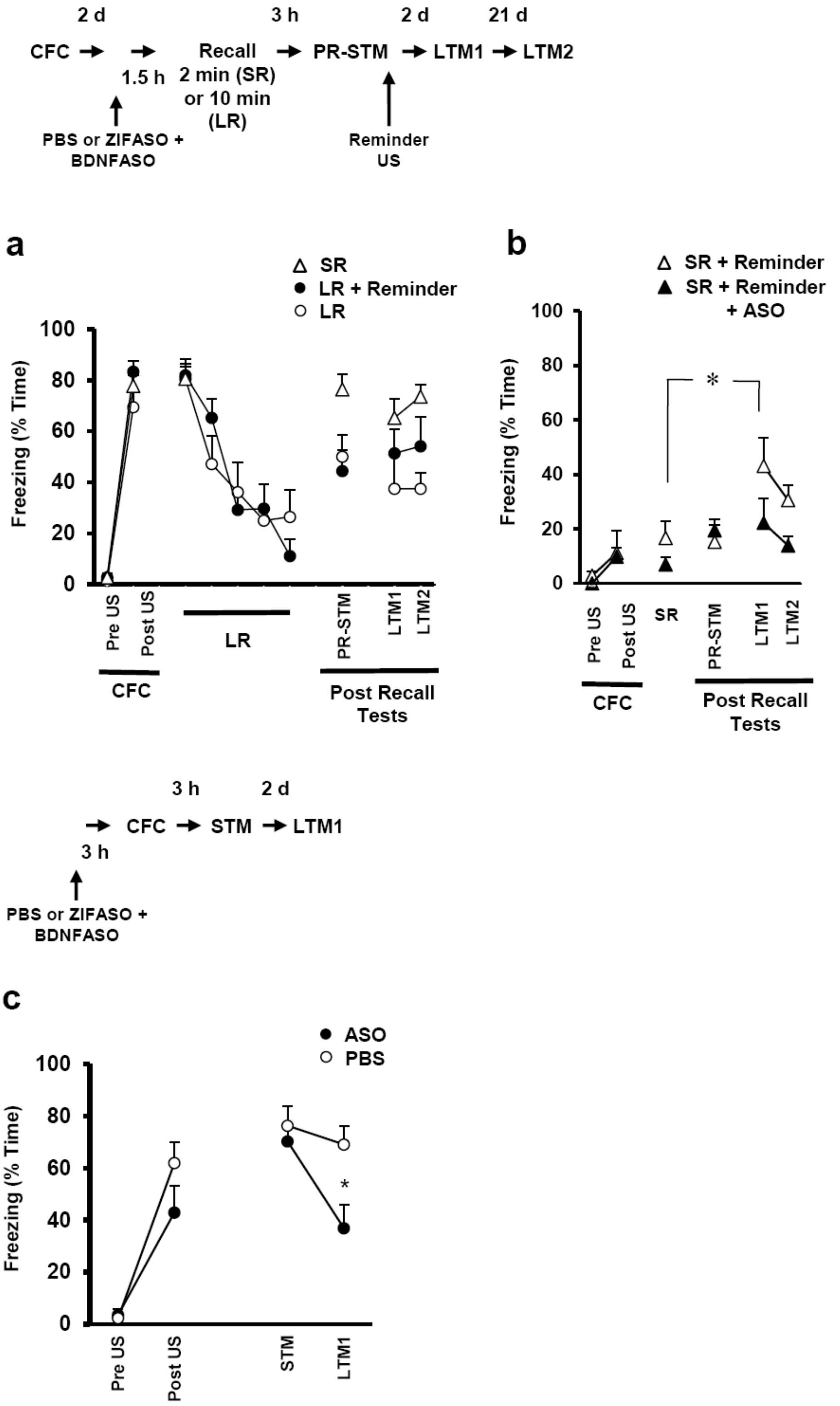


Supplementary Figure-1 Thomas

**Supplementary Figure 1**. **The expression of conditioned fear after prolonged conditioned context can be reinstated by exposure to a reminder US that causes minimal CFC.** (**a**) Long 10 min re-exposure to the conditioned context (LR) 2 d after CFC produced a within-session reduction in CR ($F_{(3.342, 33.416)}$ = 31.998, P = 0.000, ε = 0.835, repeated measures ANOVA). At the PR-STM test 3 h later, LR groups showed lower CR than those rats that underwent 2 min recall (SR group). The reduction of CR in the LR group was maintained at tests LTM1 and LTM2 2 and 23 d later ($P_{(1, 15)} < 0.05$, Tukey's test), but not in the LR + Reminder group that received a mild 2 s, 0.25 mA reminder US at the end of the PR-STM ($P_{(1, 15)}$ = 0.217, Tukey's test). (**b**) Two groups of rats were trained using a mild 2 s, 0.25 mA US, with one group receiving combined BDNFASO + ZIFASO infusions 1.5 h before SR 2 d later. There were small increases in freezing behavior during training and recall ($F_{(1.432, 14.321)}$ = 4.383, P = 0.026, ε = 0.716, repeated measures ANOVA), but no differences between the groups (TEST X INFUSION; $F_{(3.342, 33.416)}$ = 0.685, P = 0.515, ε = 0.835, repeated measures ANOVA). The reminder was able to further increase the CR, but not after combined BDNFASO + ZIFASO infusions. n = 6/group. * P < 0.05 compared to SR and LTM1. (**c**) ASO is effective at a 3 h delay between intrahippocampal infusion and CFC using a strong unconditioned stimulus. Combined BDNFASO and ZIFASO infusions or PBS were given 3 h prior to CFC using a strong 2 s, 0.5 mA US, followed by a STM and LTM1 test 3 h and 2 d later. There was no effect of combined ASO infusions on CFC (TEST X INFUSION; $F_{(1, 12)}$ = 2.871, P = 0.116, repeated measures ANOVA). However, group differences at STM and LTM1 (TEST X INFUSION; $F_{(1, 12)}$ = 7.563, P = 0.018, repeated measures ANOVA between STM and LTM1) were measured, which manifested as a decrease in conditioned fear behavior at LTM1 in the ASO-infused rats. Therefore, combined BDNFASO and ZIFASO infusions given 3 h before CFC are effective at preventing *de novo* conditioning, with no significant effects on freezing behavior at Post US and STM sessions. n = 6/group, except for (**c**) where n=7/group. *P < 0.05, one way ANOVA. Results are shown as Mean ± SEM.

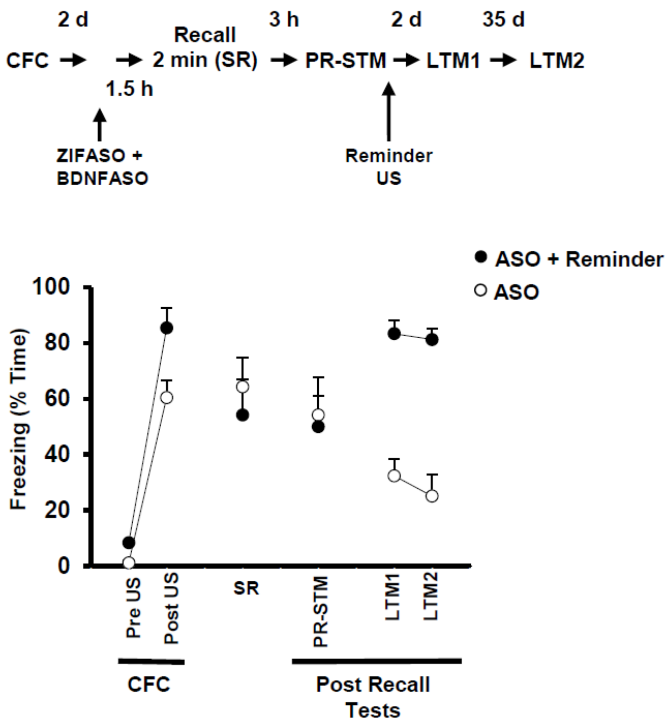


Supplementary Figure-2 Thomas

**Supplementary Figure 2**. **Reinstatement of the CR by a reminder US is seen when both ZIFASO and BDNFASO are co-infused before the recall of CFM.** There was no difference between groups of rats trained using the standard 2 s, 0.5 mA US during CFC and at SR (TEST X INFUSION; $F_{(1.505, 15.054)} = 2.907$, $P = 0.078$, $\varepsilon = 0.753$, repeated measures ANOVA. Co-infusion of BDNFASO and ZIFASO before SR, resulted in a sustained and marked reduction in conditioned freezing at LTM1 and LTM2 tests. However, this impairment was not seen in a similarly infused group given a 2 s, 0.25 mA reminder US at the end of the PR-STM (TEST X INFUSION; $F_{(1.405, 14.05)} = 8.212$, $P = 0.008$, $\varepsilon = 0.702$, repeated measures ANOVA). This indicates that the impairment of conditioned fear caused by the intrahippocampal administration of BDNFASO and ZIFASO before short CFM recall is reversed by the imposition of a reminder US. n = 8/group. Results are shown as the Means ± SEM.

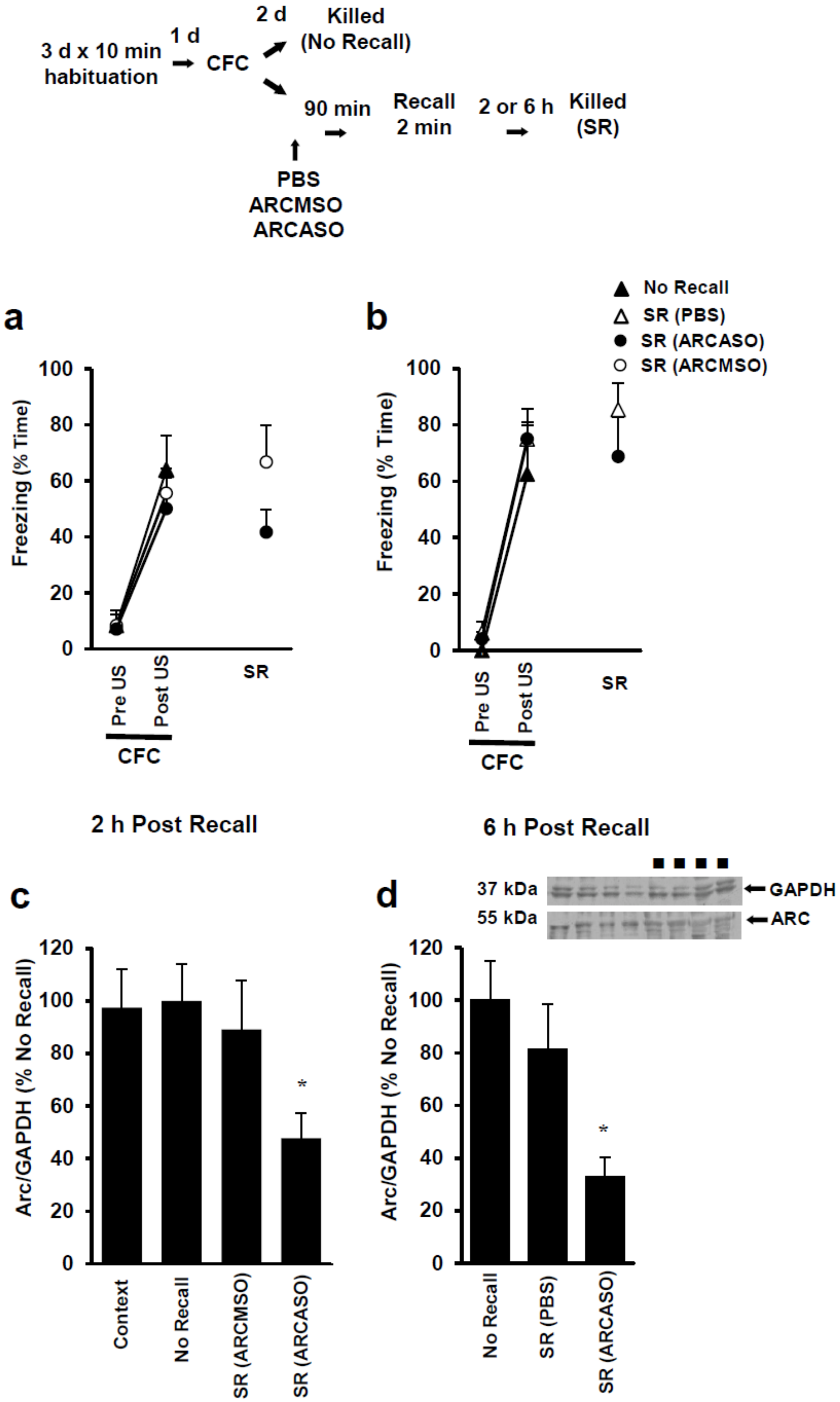


Supplementary Figure-3 Thomas

**Supplementary Figure 3**. **Intrahippocampal ARCASO infusions before the short recall of CFM produce a long-lasting reduction of Arc in the CA1**. There were no differences in the CR of the rats assigned to the different experimental and control groups to measure Arc levels either (**a**) 2 h after SR ($F_{(1, 15)} = 0.496$, $P = 0.618$, repeated measures ANOVA), or (**b**) 6 h after SR ($F_{(1, 9)} = 0.121$, $P = 0.887$, repeated measures ANOVA). Rats undergoing recall showed robust conditioned freezing during SR, with no significant differences between ARCASO and control ARCMSO (SR ARCMSO) or PBS (SR PBS) groups, infused 90 min before recall. Decreased levels of Arc levels in CA1 were seen (**c**) 2 h and (**d**) 6 h after SR in the ARCASO, but not ARCMSO or PBS-infused, animals compared those that were CFC but did not undergo recall (No Recall) (*$P < 0.05$, Tukey's test). The levels of Arc in CA1 were the same in the No Recall group and in those animals that were not conditioned but only habituated to the context only (Context). n = 6 (2 h group) and n = 4 (6 h group). ■ = Representative levels of protein in individual ARCASO samples compared to PBS-infused controls. Results are shown as the Means ± SEM.

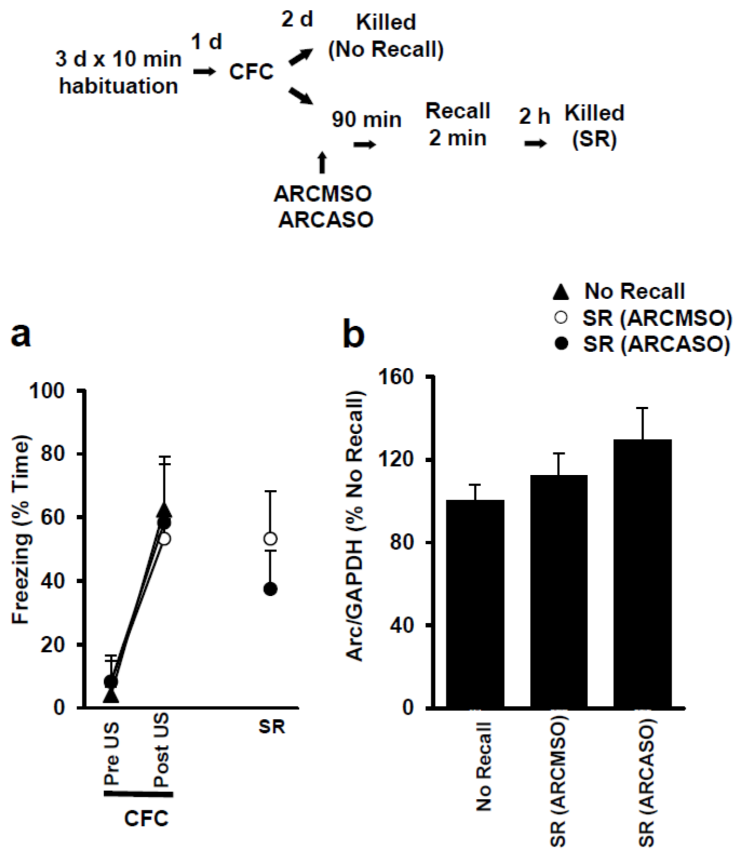


Supplementary Figure-4 Thomas

**Supplementary Figure 4**. **Intrahippocampal ARCASO infusions before recall of CFM have no effect on Arc protein in the dg/CA3**. (**a**) There were no differences in the behavioral responses Pre US or Post US of the rats in the SR (ARCASO), SR (ARCMSO) or the No Recall control groups during CFC ($F_{(1, 15)}$ = 0.335, P = 0.723, repeated measures ANOVA). Further, there were no differences in the conditioned freezing behavior in the SR (ARCMSO) and SR (ARCASO) rats during the SR recall session 2 days later. (**b**) The levels of Arc in dg/CA3 2 h after recall were the same in all three groups. n = 4 for No Recall and SR (ARCASO) groups and n = 5 for the SR (ARCMSO) group. Results are shown as the Mean ± SEM.

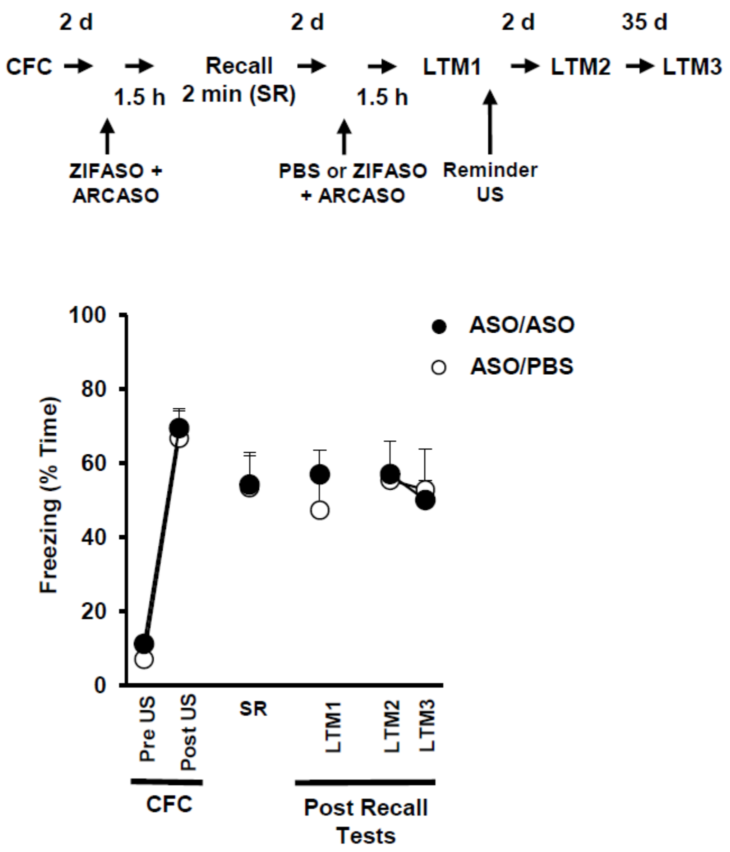


Supplementary Figure-5 Thomas

**Supplementary Figure 5. Co-infusion of ZIFASO and ARCASO do not result in an impairment of reconsolidation.** During CFC there were no differences in the freezing responses between the rats receiving co-infusions of ZIFASO and ARCASO before SR compared with PBS-infused animals (TEST X INFUSION; $F_{(1,10)} = 0.029$, $P = 0.867$, repeated measures ANOVA). Robust levels of conditioned freezing at SR and in subsequent LTM tests were seen in both groups (TEST X INFUSION; $F_{(2.78,27.801)} = 0.358$, $P = 0.784$, $\varepsilon = 0.927$, repeated measures ANOVA). Therefore, we observe no reduction in CR at LTM tests after the co-infusion of ZIFASO and ARCASO, in contrast to the effects of singular infusions. Since the retrieval of the CFM is intact, this abrogates the interpretation that the combined infusions of ASO non-specifically affect hippocampal function. n = 6/group. Results are shown as the Mean ± SEM.

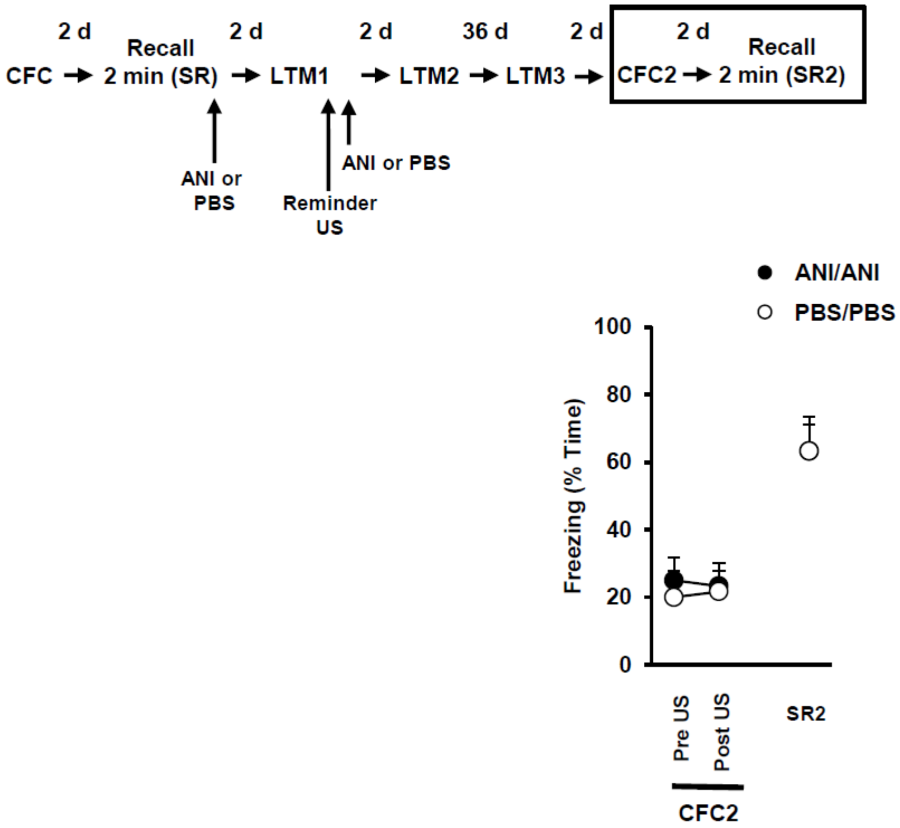


Supplementary Figure-6 Thomas

**Supplementary Figure 6. Fear conditioning in a new context is intact following anisomycin treatment.** All CFC rats from the ANI/ANI and PBS/PBS groups (see Figure 3 main text) were fear conditioned in a second novel context (CFC2), 2 days after their last LTM test (LTM3). Pre US, the rats showed a low level of freezing responses indicating that the rats could discriminate between the conditioned context and the novel context. There was an overall effect of test (TEST; $F_{(1.684, 6.736)} = 17.43$, $P = 0.000$, $\varepsilon = 0.842$, repeated measures ANOVA), although no interactions of test with ANI treatment (TEST X INFUSION; $F_{(1.684, 6.736)} = 0.051$, $P = 0.951$, $\varepsilon = 0.842$, repeated measures ANOVA) or between group differences of ANI treatment (INFUSION; $F_{(1.684, 6.736)} = 0.152$, $P = 0.707$, $\varepsilon = 0.842$, repeated measures ANOVA) were observed. At a 2 min recall test (SR2), all animals showed a robust conditioned fear response with no between group differences (INFUSION; $F_{(1, 8)} = 0.000$, $P = 1.000$, one-way ANOVA at SR2). n = 5/group. Results are shown as the Mean ± SEM.
